# Mesoporous Silica-Based Nanoplatforms Are Theranostic Agents for the Treatment of Inflammatory Disorders

**DOI:** 10.3390/pharmaceutics15020439

**Published:** 2023-01-28

**Authors:** Bhagavathi Sundaram Sivamaruthi, Subramanian Thangaleela, Periyanaina Kesika, Natarajan Suganthy, Chaiyavat Chaiyasut

**Affiliations:** 1 Office of Research Administration, Chiang Mai University, Chiang Mai 50200, Thailand; 2 Innovation Center for Holistic Health, Nutraceuticals, and Cosmeceuticals, Faculty of Pharmacy, Chiang Mai University, Chiang Mai 50200, Thailand; 3 Bionanomaterials Research Laboratory, Department of Nanoscience and Technology, Alagappa University, Karaikudi 630003, India

**Keywords:** nanoparticles, mesoporous silica nanoparticles, inflammatory diseases, neuroinflammation, nanomedicine, drug delivery

## Abstract

Complete recovery from infection, sepsis, injury, or trauma requires a vigorous response called inflammation. Inflammatory responses are essential in balancing tissue homeostasis to protect the tissue or resolve harmful stimuli and initiate the healing process. Identifying pathologically important inflammatory stimuli is important for a better understanding of the immune pathways, mechanisms of inflammatory diseases and organ dysfunctions, and inflammatory biomarkers and for developing therapeutic targets for inflammatory diseases. Nanoparticles are an efficient medical tool for diagnosing, preventing, and treating various diseases due to their interactions with biological molecules. Nanoparticles are unique in diagnosis and therapy in that they do not affect the surroundings or show toxicity. Modern medicine has undergone further development with nanoscale materials providing advanced experimentation, clinical use, and applications. Nanoparticle use in imaging, drug delivery, and treatment is growing rapidly owing to their spectacular accuracy, bioavailability, and cellular permeability. Mesoporous silica nanoparticles (MSNs) play a significant role in nano therapy with several advantages such as easy synthesis, loading, controllability, bioavailability over various surfaces, functionalization, and biocompatibility. MSNs can be used as theranostics in immune-modulatory nano systems to diagnose and treat inflammatory diseases. The application of MSNs in the preparation of drug-delivery systems has been steadily increasing in recent decades. Several preclinical studies suggest that an MSN-mediated drug-delivery system could aid in treating inflammatory diseases. This review explains the role of nanoparticles in medicine, synthesis, and functional properties of mesoporous silica nanoparticles and their therapeutic role against various inflammatory diseases.

## 1. Introduction

Inflammatory responses are a series of complex interactions that emerge from stimuli such as injury and exposure to allergens, toxins, chemicals, irradiation, and pathogens. The affected or damaged tissues undergo repair procedures that recruit different types of immune cells and pro- and anti-inflammatory mediators [1]. Various diseases such as asthma, arthritis, atherosclerosis, autoimmune disease, and cancer may occur due to chronic inflammation [2].

Neuroinflammation is a significant inflammatory response within the central nervous system (CNS) and a hallmark sign of neurodegenerative diseases (NDs) such as Parkinson’s disease (PD), amyotrophic lateral sclerosis (ALS), Alzheimer’s disease (AD), multiple sclerosis (MS), neuromyelitis optical spectrum disorders (NMOSD), and autoimmune encephalitis [3,4]. Anti-inflammatory therapies target different inflammatory cytokines such as IL-1, IL-6, IL-18, IFN β, and TNF-α in various immune diseases [3]. 

**Figure 1:**
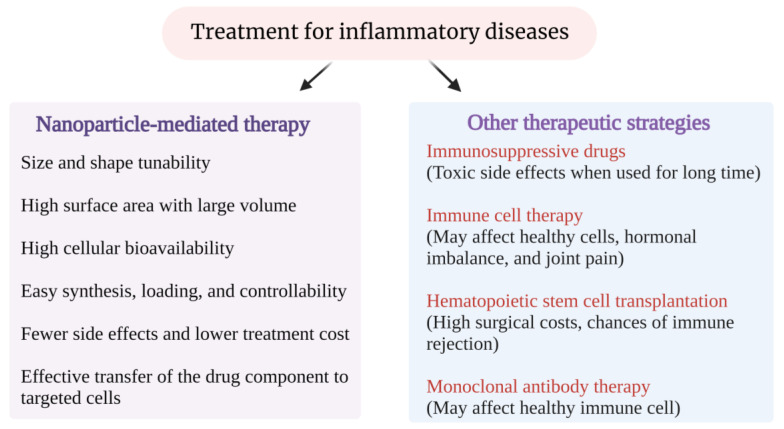
The advantages of NP-mediated therapeutic technologies to treat inflammatory diseases.

Nanoparticle (NP)-mediated therapeutic technologies presume effective and promising drug delivery methods for inflammatory diseases [5] [Figure 1].

Based on their origin, NPs can be categorised into natural and engineered (artificially designed) NPs. Natural-type NPs are found in the natural environment and produced in volcanic smoke, from the erosion of geological materials, by degradation of biological materials and vegetable residues, and through combustion. The engineered NPs are synthesized in an aggregated manner, such as carbon nanotubes, fullerenes, quantum dots, and nano fibres [6]. NPs can be tagged with designated antibodies, aptamers, and peptides and can be delivered to the targeted cells with fewer limitations and high specificity [7]. Various NPs have been developed as drug-delivery platforms; the mesoporous silica nanoparticles (MSNs) play a significant role because of their ease of administration, controllability, tenability, and biocompatibility. MSNs are widely employed as photosensitizer drug carriers. MSNs are organically modified silica with mesopores to provide a wide surface area to hold the drug material prominently employed in photosensitizer delivery systems [8].

### 1.1. Nanomaterial-Based Drug Delivery Systems

Advancements in nanotechnology in the era of biomedical application have helped overcome the obstacles encountered in the therapy and diagnostics of serious human ailments. Nanotechnology-based drug-delivery systems (DDS) facilitate targeted drug delivery, minimize the drug dosage and frequency, enhance the circulation period, and reduce the adverse side effects, thereby improving bioavailability and therapeutic efficiency. Several organic and inorganic nanocarriers such as metal/metal oxide-, polymer-, and lipid-based carriers have been developed as DDS, but have certain limitations such as structural complexity and poor solubility and bioavailability. 

In recent decades porous materials, which are low-density solids having distinct pore structures with a larger surface area, high selectivity and permeability, and low refractive coefficient, have received much focus in various sectors such as energy storage, biomedicine, transportation, and catalysis [9,10]. Porous materials allow both internal and external surface functionalization. The interaction of atoms/ions in the walls of porous materials with the drug facilitates controlled loading and release [11]. Porous material can be organic (carbon nanotubes and nanowires) or inorganic (metal/metal oxide, quantum dots, and hybrid composite). Stability, resistance to microorganisms, and biocompatibility are the reasons for selecting inorganic nanoparticles for several applications [12].

The International Union of Pure and Applied Chemistry (IUPAC) classifies porous materials by size. Microporous, mesoporous, and macroporous materials are 2 nm, 2 to 50 nm, and greater than 50 nm in size, respectively [13]. Among these porous nanomaterials, mesoporous materials were promising candidates for catalysis, fabrication, and medicine, specifically for drug delivery, owing to their unique physiochemical characteristics, such as larger surface areas, tunable pores, and excellent mechanical and thermal stability. The discovery of silica nanoparticles helped to overcome the limitations of currently used nanomaterials, such as poor solubility and bioavailability. It provoked its use in porous glass and disordered silica gels for drug-delivery applications [14].

### 1.2. Nanoparticles and Their Role in Medicine

The use of NPs in the medical field is of great realistic value due to their shape, stability, charge, and binding capacity [15]. Nanomaterial usage in the medicinal field attains more improvements in modifying basic biological parameters such as solubility, immunogenicity, diffusivity, shelf-life in the blood, and active drug release rate.

Cancer is a deadly disease caused by uncontrolled and random cell division with high invasiveness. NPs are used in cancer treatment to deliver cancer drugs to tumor sites with improved drug stability, efficient targeting, and no side effects [16]. Liposomes tagged with doxorubicin were more efficient against metastatic breast cancer cells [17]. Liposomes, dendrimers, and hydrogels are organic NPs used in brain cancer treatment [18]. Fe_3_O_4_ and gold NPs, quantum dots, and polymers are inorganic NPs used as nanocarriers for targeting and delivering drugs in brain cancer treatment [19]. The Food and Drug Administration (FDA) confirmed that daunorubicin, doxorubicin liposomal, cytarabine liposomal, pegaspargase, and nab-paclitaxel (nab: active ingredient of nanoparticle, albumin-bound) are efficient drugs against various cancers, including breast, ovarian, renal, and lung cancer [20]. Breast, ovarian, and lung cancers have been treated with NPs [21]. 

Due to the clinical limitations of conventional treatment methods, the nanoparticles exert their unique effects in skeletal tissue engineering. Magnetic iron oxide NPs (MION) are used in tissue-regeneration studies and real-time visualization for tracking the transplanted cells, cell growth, and activation of ion channels [22]. The gene therapy approach is useful in bone repair and regeneration. Calcium phosphate NPs (CaNPs) have been used as non-viral vectors in RNAi delivery to regulate gene expression in the microenvironment of bones. CaNPs are cellular-friendly in maintaining biocompatibility, osteoconductivity, osteoinductivity, and intact affinity for nucleic acid binding [23]. 

NP types, such as polymeric, solid lipid, quantum dots, and liposomes, are conjugated with various drugs to cross the blood-brain barrier (BBB) and target lesions and biomarkers without side effects [24]. Poly (butyl cyanoacrylate) nanoparticles (PBCA) with thioflavins were used to target amyloid β fibrils in the murine AD model [25]. NPs composed of polyethylene glycol (PEG) and poly hexadecyl cyanoacrylate (PHDCA) have more adaptability in the cellular environment. Since PEG is not recognized as a foreign material in the host system, phagocytes, their increased shelf life along with drug carriers in the bloodstream, and the combination of PEG with PHDCA increase the ability to cross the BBB [26]. These advantages validate the combination of PEG and other drug carriers as an eminent nanocomposite to treat neurological disorders. PEG also can be conjugated with liposome-mediated nano drug delivery or gene delivery across the BBB. This approach has been practiced against chronic neurological diseases such as Huntington’s and Rett’s syndrome [27]. 

Nano-drug-delivery technologies have created a huge impact on respiratory disease treatment. Respiratory diseases such as lung cancer, asthma, chronic obstructive pulmonary disease (COPD), and even pulmonary tuberculosis can be treated with the help of NPs with enhanced drug targeting. Carbon nanodots, steroid nanoparticles, salbutamol, liposome-mediated NPs, and gold solid-liquid NPs are now used to treat respiratory disorders [28].

NPs have been used against inflammatory diseases as targeted drug-delivery systems. NPs conjugated with anti-inflammatory drugs favourably modulate inflammatory and vascular cell functions [29]. Among inflammatory diseases, neuroinflammation is the main pathological sign of neurodegenerative diseases. The utmost challenge in treating CNS inflammation is bypassing the natural BBB. NPs serve an important role in delivering drugs into the microglial cells and are also helpful in targeting, tracing, diagnosing, or sensing the specific site of action. NPs are beneficial in transporting anti-inflammatory drugs across the BBB and effective due to their biodegradability and biocompatibility properties [30]. 

The present study provides an overview of the types, synthesis, mechanisms of formation, properties, and functionalization of MSNs, and emphasizes MSNs as a theranostic tool for inflammatory diseases.

## 2. Methods

**Figure 2:**
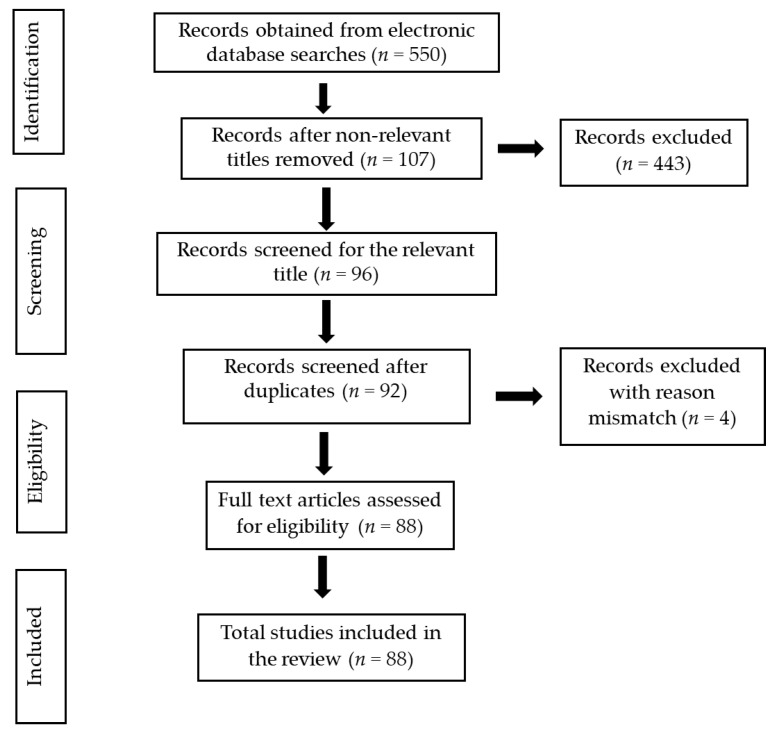
Schematic representation of the PRISMA chart explaining the selection of studies.

Documents were searched from scientific databases such as the Scopus, PubMed Central, Web of Science, and Medline platforms. The keywords used for searching were “inflammatory diseases”, “inflammation”, “neuroinflammation”, “nanoparticles in medicine”, “mesoporous silica nanoparticles”, “synthesis of mesoporous silica nanoparticles”, “properties of mesoporous silica nanoparticles”, “function of mesoporous silica nanoparticles”, “mesoporous silica nanoparticles as drug delivery system”, “role of mesoporous silica nanoparticles in inflammatory diseases”, and “nanoparticles and inflammatory diseases”. The research and review articles and abstracts related to our topic of interest were examined and chosen for the preparation of the manuscript. Abstracts and research papers in the English language were used in the present review. The PRISMA (Preferred Reporting Items for Systematic Reviews and Meta-Analyses) chart describes the selection criteria of the collected articles (Figure 2).

## 3. Mesoporous Silica Nanoparticles (MSN)

MSNs are a porous inorganic framework synthesized using the inorganic silica sources sodium silicates or silica tetraethyl orthosilicate as a precursor with quaternary ammonium salts as the surfactant. The size and porosity of MSNs are regulated by various factors such as surfactants, the source of silica, ion strength, aging duration, temperature, and pH [31]. Highly ordered mesoporous silica came into the limelight after the fabrication of the silica-based material Mobile crystalline material 41 (MCM) by Kazuyuki Kuroda’s groups and scientists at Mobil Oil Corporation from aluminium silicate gel [32]. MSNs have a unique porous solid framework with a large surface area facilitating functionalization with various functional groups for targeted drug delivery. MCM-41, MCM-48, and SBA-15 are the most common mesoporous silica materials with two-dimensional hexagonal and three-dimensional cubic structures possessing pore sizes of 2–10 nm.

The unique mesoporous structure with high chemical stability, variable pore size, larger surface area, ease of outer surface functionalization, biocompatibility, and sustained release of entrapped materials makes this nanomaterial highly attractive as a drug-delivery system, diagnostic agent, and sensing and separating agent [33]. Quick cellular internalization of MSN in plant and animal cells without any toxic effect is another unique feature of functionalized MSN. As silica-based materials are inert and non-toxic, FDA approves them as safe to construct nanoplatforms for delivering drugs and cancer chemotherapy [34].

### 3.1. Types of MSNs

**Figure 3:**
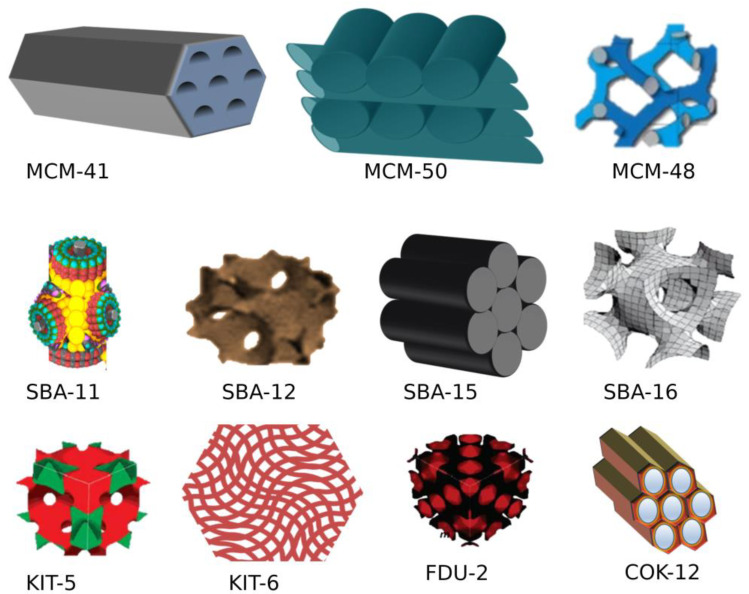
Types of mesoporous silica based on shape and morphology. (Figure created using BioRender.com; accessed on 2 December 2022).

Different forms of crystalline MSN are formed by altering the cationic surfactant to silica precursor ratio at basic conditions (pH 8–12). If this ratio is less than 1, then hexagonal two-dimensional mobile crystalline material 41 (2D MCM 41) is formed, and if the ratio is greater than 1, three-dimensional cubic MCM-48 is formed. The increase in the ratio leads to 3D lamellar MCM-50 [35]. Other mesostructured materials such as Santa Barbara Amorphous-11 (SBA-11), SBA-12, and SBA-15 were synthesized using poly (alkylene oxide) and alkyl poly (ethylene oxide) (PEO) oligomeric surfactants as templates [36]. FSM-16, folded sheets of mesoporous materials, were produced using quaternary ammonium surfactant and layered polysilicate kanemite as template and adsorbent, respectively, via catalysis. Several other MSNs of varying pore size, shape, and symmetry were fabricated and termed Technical Delft University (TUD-1), Hiroshima Mesoporous Material-33 (HMM-33), and Centrum voor Oppervlaktechemie en Katalyse/Centre for Research Chemistry and Catalysis (COK-12). MCM-41, MCM-50, SBA-15, and SBA-16 are the commonly used MSNs for drug delivery [37]. Figure 3 and Table 1 represent the types of MSN with their unique properties widely used for drug delivery.

### 3.2. Fabrication of MSNs

Stober was a pioneer in synthesizing nanosized silica particles from micron-sized silica; hence it was termed Stober’s method. By manipulating the reaction parameters, MSNs of different sizes, morphology, and pore size were fabricated. Grun et al. modified Stober’s method for synthesizing spherical-shaped MCM-41. As pore size and volume play a key role in DDS, they can be controlled by altering the pH, temperature, surfactant concentration, and silica precursor. The components required to synthesize MSNs are an inorganic silica source/precursor, surfactant, template, and catalyst [46].

Predecessor: The most widely used silica precursors for MSN synthesis include glycerol-derived polyol-based silanes, orthosilicic acid, sodium metasilicate, tetraethyl orthosilicate (TEOS) or tetramethoxysilane (TMOS), Tetra methoxy vinyl silane (TMVS), and tetrakis (2-hydroxyethyl) orthosilicate (THEOS). TEOS and TMOS are poorly soluble and pH-dependent, replaced by glycerol-derived polyol-based silane precursors. MSNs of different shapes such as spherical, rod, and hexagonal tubular shapes can be obtained using 3-aminopropyltrimethoxysilane (APTMS), 3-[2-(2-aminoethyl amino) ethylamino] propyltrimethoxysilane (AEPTMS), ureidopropyltrimethoxysilane (UDPTMS), 3-isocyanatopropyltriethoxysilane (ICPTES), 3-cyanopropyltriethoxysilane (CPTES), and allyltrimethoxysilane (ALTMS) as organoalkoxysilanes precursor [47].

Surfactant: These are classified as below, based on the surface charges [48,49].

- Cationic surfactants: Surfactants such as cetyl trimethyl ammonium chloride (CTAC), cetyl pyridinium chloride (CPyC), and cetyl tri methyl ammonium bromide (CTAB) with positively charged hydrophilic trimethyl ammonium/pyridinium groups as polar head and a sixteen-carbon hydrocarbon chain nonpolar tail act as a cationic surfactant.
- Anionic surfactant: Low-cost, eco-friendly anionic surfactants such as sodium salts of alkyl carboxylic acids, phosphoric acid, and sulfonic acid with a negatively charged polar head and hydrocarbon nonpolar tail are widely used as an anionic surfactant.
- Non-ionic surfactants: Non-ionic triblock copolymers such as alkyl poly (ethylene oxide) (PEO) oligomeric surfactants, poly (alkylene oxide) block copolymers, Triton X-100, polysorbate, and pluronic F 127 with a non-dissociable hydrophilic head which cannot ionize in aqueous solution act as a non-ionic surfactant.
- Amphoteric/zwitterionic surfactants: Includes surfactants with a net neutral charge such as sodium dodecyl benzene sulfonate (SDBS), sodium dodecyl sulphate (SDS), phospholipids, betaines/sulfobetaine, amino acids, propyl ortho-silicate (TPOS), trimethoxy silane (TMS) and sodium metasilicate (Na_2_SiO_3_).

Catalysts: The catalyst used for the sol-gel method depends on the reaction. In an acid-catalyzed reaction, the hydrolysis is faster than the condensation reaction, which forms several small silica particles/networks of gels. In a base-catalyzed reaction, condensation is faster than hydrolysis resulting in larger silica particles/solid spheres forming. Diethanolamine (DEA), triethanolamine (TEA), hydrochloric acid, ammonia, and sodium hydroxide are commonly used as catalysts [50].

### 3.3. Mechanism of Formation of MSN

**Figure 4:**
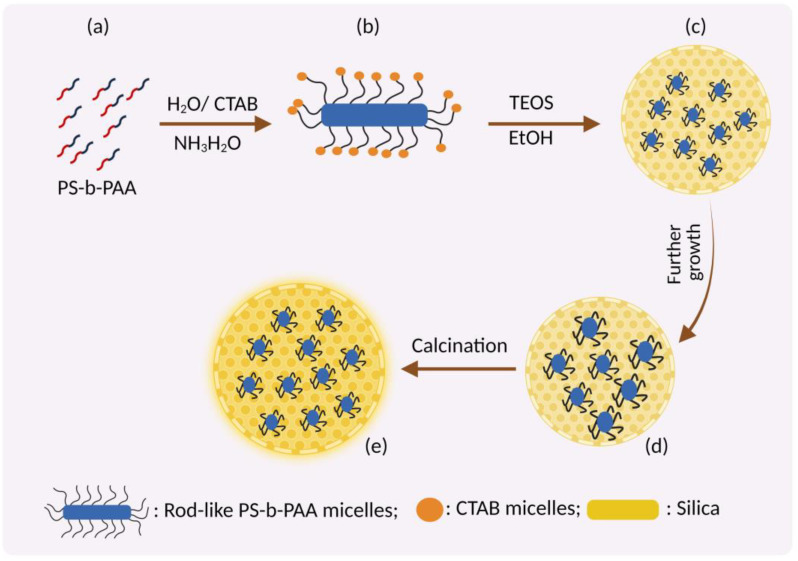
Formation of MSNs by Stober’s method. (**a**) Amphiphilic block copolymer (polystyrene-b-poly(acrylic acid), PS-b-PAA) and CTAB as co-templates in aqueous ammonia solution; (**b**) Formation of composite rod-like micelles due to electrostatic interaction between CTA+ and PAA; (**c**) Formation of core part by deposition of silicate of TEOS on rod-like micelles of CTAB-coated PS-b-PAA; (**d**) Dual mesoporous silica nanoparticles by self-assembly of TEOS with CTAB; (**e**) Calcination forming the final core-shell dual mesoporous silica spheres. (Figure created using BioRender.com; accessed on 2 December 2022).

Hydrolysis of tetramethyl orthosilicate (TMOS), the precursor to silicate ions, tends to adsorb on the surfactant micelles. When the charge around the surfactant reduces, hydrolysis starts, followed by condensation of the silica precursor. Intermicellar repulsion prevents the formation of small silica aggregates, leading to the formation of discrete hexagonally ordered mesopores of silica [51] (Figure 4).

#### 3.3.1. Swelling Shrinking Mechanism

Tetraethyl orthosilicate (TEOS) is used as a precursor without solvent. TEOS in an aqueous solution shows phase separation but, under continuous stirring, forms an emulsion-like system. The addition of TEOS to the amphibolic surfactant CTAB solubilizes in the hydrophobic core forming large micelles. TEOS, on hydrolysis, becomes hydrophilic, is released as a monomer in the aqueous surrounding, and is adsorbed to CTAB via electrostatic attraction. Consumption of TEOS from the hydrophobic core reduces micelle size. Hydrolysis and condensation occur simultaneously, forming the silica shell around the micelles. The adjacent micelles aggregate to form a mesoporous structure [52].

#### 3.3.2. Sol-Gel Method

The majority of MSN fabricated using the Stober technique undergo the sol-gel process. This process of MSN synthesis involves two stages, nucleation and growth. Nucleation involves hydrolysis of the precursor metal alkoxides TEOS in the presence of catalyst HCl/NH4OH solution to form silanol groups. Under an acidic environment, the alkoxy group (O-R) and a hydroxyl group (O-H) undergo protonation followed by subsequent deprotonation leaving an alcohol group R-OH. The growth stage involves the polymerization of silanol groups or between silanol and ethoxy groups where the -OH group of one partially hydrolyzed protonated precursor condenses with -OH groups of another partially hydrolyzed precursor molecule forming siloxane bridges (Si-O-Si), leading to the formation of oligomers and subsequent condensation, forming a mesoporous silica structure [53]. The hydrolysis and condensation of alkoxide monomers into a colloidal solution (sol) turns into an ordered polymer (gel) network. This process requires an acid or base as the catalyst. 

**Figure 5:**
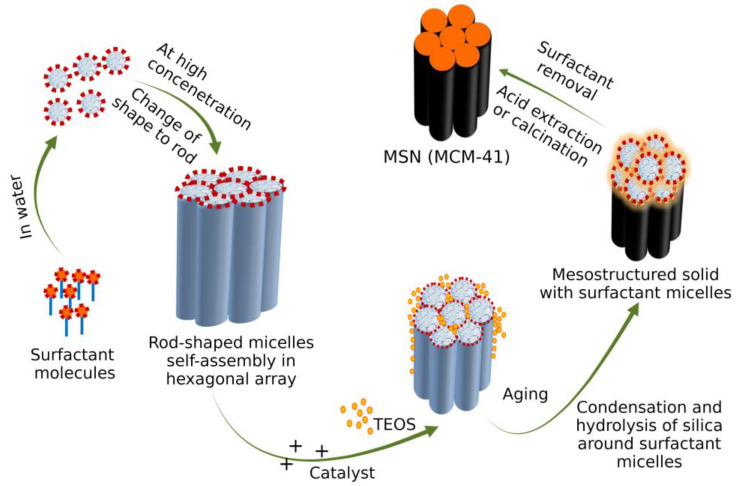
Formation of MCM-41 by sol-gel method. (Figure created using BioRender.com; accessed on 2 December 2022).

The rate of hydrolysis depends on the ratio of Si/H_2_O. Under acidic conditions, fast hydrolysis occurs, while in basic conditions, condensation occurs. Multiple condensation results in a chain-like structure in the sol and network-like structure in the gel form. The sol-gel process can be modified to yield particles of the desired size and properties. The sol-gel method was used for the synthesis of MCM-41. CTAB acts as an amphiphilic surfactant and tends to assemble as a spherical micelle in water and precursor polysilicic acid. The silica precursor interacts with the surfactant by electrostatic and hydrogen bonding, forming amorphous silica, and then molding it to a mesoporous structure (Figure 5). The surfactant is removed by repeated washing and then calcinated.

#### 3.3.3. Evaporation-Induced Self-Assembly (EISA)

**Figure 6:**
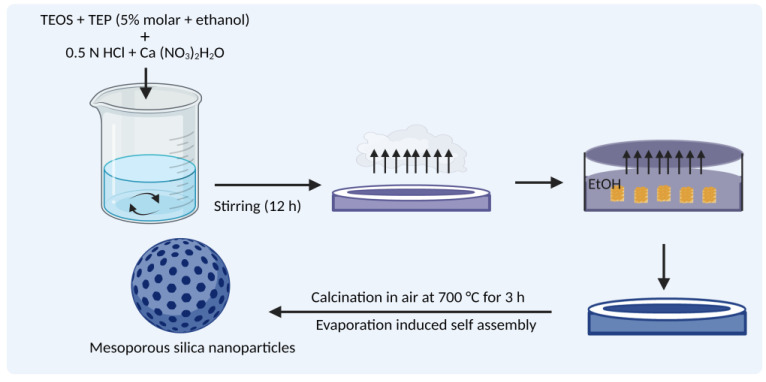
Formation of MSN by the evaporation-induced self-assembly method. (Figure created using BioRender.com; accessed on 2 December 2022).

In the process of EISA, all the reactants change their concentration during evaporation, resulting in the silica precursor’s organization as the liquid-crystal-like template [54]. The precursor formulation TEOS is prepared in ethanol/water solvent containing the surfactant CTAB which is injected into an aerosol generator with sufficient air flow and pressure to produce a monodispersed solid droplet. During the alcohol evaporation, micelle formation is induced, which converts the silica-surfactant co-assembly into liquid crystal mesophase (Figure 6).

#### 3.3.4. Removal of Surfactant after Synthesis

Removal of surfactant from the final MSN product is essential for the following reasons.

- For biomedical applications, the cytotoxic nature of surfactants affects living cells due to its interaction with the phospholipid layer of cells leading to lysis [55].
- Surfactants reduce the pore size and volume, affecting the drug-loading efficiency and release kinetics [56].
- The presence of surfactants affects surface functionalization.

Calcination: MSNs calcinated at 800 °C remove the surfactant forming hollow cylindrical MSNs. Calcination affects the surface functionalization as Si-OH bonds are converted to Si-O-Si bonds at higher temperatures, compressing the surface area and pore size. To overcome the limitation of calcination, a solvent extraction method is used to remove the surfactant [57].

Solvent extraction: The solvent used depends on the surfactant and experimental conditions.

Solvents such as water, ethanol, hydrochloric acids, ammonium nitrate, and other alcohols have been used. Although the solvent extraction method restores the pore size integrity, the complete removal of surfactant is difficult [58].

Chemical-assisted oxidation: Hydrogen peroxide is commonly used as a chemical oxidant to remove surfactants with an oxidation reaction, increasing the pore diameter with reduced pore volume and surface area. The number of silanol groups on the surface of MSNs is higher than in calcinated samples. Oxidants used are H_2_O_2_, ozone, KMnO_4_–H_2_O_2_, and NH_4_ClO_4_ [59].

Microwave digestion: Removal of surfactant is performed by exposing MSNs in HNO_3_-H_2_O_2_ solution or hexane and ethanol to microwaves for 2 min. This process increases the silanol groups with higher pore size, volume, and large surface area [60].

#### 3.3.5. Factors That Influence MSN Particle Synthesis

The following factors influence MSNs particle synthesis [61].

(a) Rate of silica interaction and condensation
(b) Assembly kinetics, nucleation, and growth rates
(c) Charge of silica: The rate of silane hydrolysis and condensation of the siloxane bond depends strongly on the charge states.
(d) pH of reaction mixture: The silica charge depends on the reaction mixture’s pH. Hydrolysis of the Si–OR bond in silanes occurs faster in an acidic/basic environment compared to a neutral solution. At a pH below the isoelectric point (IEP of silica—2.0), the silica is positively charged, and its charge density increases with a decrease in pH. In reaction mixtures with a pH above the IEP, the silica becomes negatively charged and the charge density increases with the pH. At pH 2–4, the negatively charged silicates interact with positively charged surfactants via electrostatic and hydrogen bond interactions. At pH 4–7, the negative charge density of silicate increases; hence interaction with the surfactant occurs only through the electrostatic force [62].

Silica condensation reaction:

≡Si-OH + HO-Si≡ ⇌ ≡Si-O-Si≡ + HOH

≡Si-OH + RO-Si≡ ⇌ ≡Si-O-Si≡ + ROH

where R represents methyl, ethyl, propyl, and butyl groups, etc.

The condensation rate increases with an increase in the negative charge of silicate due to its nucleophilic attack.

(e) Co-surfactants used: Alcohols such as ethanol and butanol influence the pore size, shape, and flexibility when their concentration increases. An increase in co-surfactant concentration disrupts the spherical shape of MSNs, forming amorphous particles with disordered pore sizes.
(f) Solvent used: Alcohols such as ethanol, propanol, butanol, and pentanol enhance the pore formation in mesopores. The channel rotations of mesoporous materials are also modified by alcohol. Removal of surfactants after the synthesis of MSNs is promoted by alcohol with a high boiling point which prevents aggregation of MSNs. Long-chain alcohols help MSNs transit from one phase to another.
(g) Silica sources: Sodium silicates, colloidal solutions, and organosilanes form mesoporous silicate structures more rapidly than other precursors [63].
(h) Temperature: The critical temperature for determining the final properties of MSNs is between 10 and 130 °C, within which 25 °C is the optimum temperature [64].

#### 3.3.6. Functionalization of MSNs

Despite its unique physiochemical properties, the functionalization of MSNs is required to improve their physical and chemical properties to enhance their drug adsorption and sustained release at the target site [65]. The unique architecture of MSNs facilitates functionalization, i.e., introducing various moieties both in the internal and external surface of MSN, which plays a crucial role in targeted drug delivery. MSNs have two functional surfaces: a cylindrical pore surface and an exterior particle surface. The surface-containing silanol group can be selectively functionalized for controlled drug loading and targeted cell-specific drug release [66]. The organic silanes vinyltriethoxy silane (VTES), 3-aminopropyl triethoxysilane (APTES), methoxy-PEG-silane, and 3-mercaptopropyl trimethoxysilane make the fabricated MSNs versatile and promising candidates to perform a specific task. 

Various surface-modifying functional groups include carboxyl groups, polymers such as PEG, phospholipids, polyethyleneimine (PEI), phospholipids, organic phosphates, and thiols. For the modification of the negatively charged surface, polymers such as 3-aminopropyltriethoxysilane, polylysines, and polyethyleneimine are used [67,68]. Diethoxydimethyl silane, trimethylchlorosilane, and polymethyl hydrosiloxane reduce the hydrophobicity of MSNs and improves the loading efficiency of hydrophobic drugs [69]. Generally, MSNs are functionalized in the internal/external surfaces, walls of pores, and their entry site. Entrapment of drugs with MSNs is based on various interactive forces such as covalent and hydrogen bonding, van der Waals interaction, and electrostatic forces depending on the functional group present at that site [70,71].

Functionalization influences the MSN’s behavior in biological fluids, such as solubility, dispersibility, drug loading, and drug-release kinetics. The surface modification of MSNs is carried out by co-condensation and post-synthetic modification. Co-condensation involves incorporating functional groups with silica precursors during the synthesis of MSNs. In contrast, the post-synthetic approach involves selectively attaching functional groups to the external and pore surfaces. Co-condensation has several advantages, including the wide range of organoalkoxysilanes that can be used, uniform homogenous distribution of functional groups, and high payload of the functional group without any adverse effect on the MSN structure [72]. 

Covalent attachment of ligands and imaging agents to MSNs will modify the characteristics of MSNs. For example, incorporating iron oxide into MSNs enhances the magnetic resonance imaging (MRI) properties and their therapeutic output [73]. For the delivery of hydrophobic drugs, the MSN surface is functionalized with hydrophobic groups, as these drugs cannot interact with the hydrophilic surface of MSNs. Delivery of nucleic acids is facilitated by functionalizing the surface of MSNs with the positively charged polymer PEI so that DNA/RNA can bind to the MSNs via the electrostatic force of attraction. This nonviral mode of gene delivery prevents enzymatic degradation of nucleic acids [74]. Functionalization of MSNs with a cationic polymer facilitates the delivery of antibiotics as MSNs interact with negatively charged bacterial cell walls and biofilms and quickly enter microbial cells [75]. MSNs can be conjugated with several ligands such as aptamers, growth factors, and vitamins, facilitating cellular entry by receptor-mediated endocytosis forming endosomes resulting in targeted drug delivery with reduced toxicity [76]. Incorporating photosensitizers into MSNs functionalized with specific ligands to cancer cells promotes photodynamic therapy for killing cancer cells more efficiently than radio- and chemotherapies [77]. Peripheral functionalization of MSNs facilitates the colloidal nature, chemical stability, specific targeting, and pore-gating capacity, improving the MSN’s biocompatibility and nontoxicity. 

**Figure 7:**
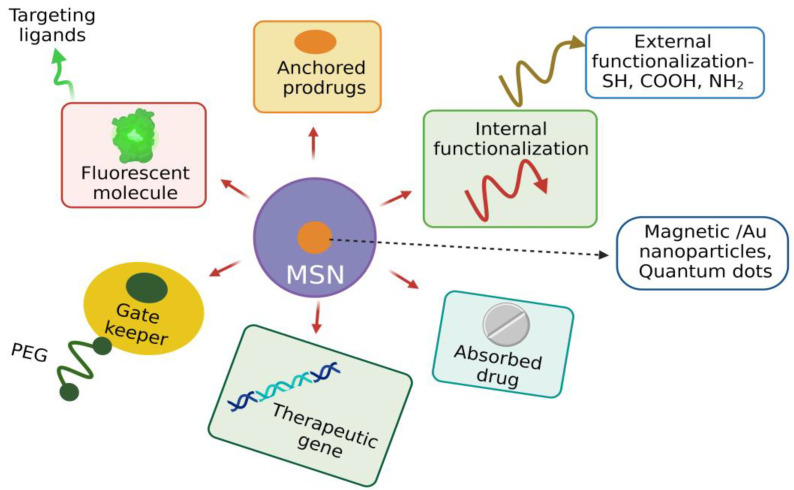
Functionalization of MSN as a promising theranostic agent enhancing its biocompatibility. (Figure created using BioRender.com; accessed on 2 December 2022).

In addition, surface functionalization promotes the co-delivery of cargo with different properties for the synergistic effect to enhance the therapeutic response of the drug. For example, MSNs coated with PEI and loaded with osteostatin functionalized with SOST siRNA effectively treat osteoporosis [78]. Surface functionalization of MSNs with various moieties based on different therapeutic strategies enhances its application efficiency (Figure 7).

### 3.4. Unique Structural Features of MSNs Suitable for Biomedical Applications

#### 3.4.1. Ordered and Tunable Pore Structure

MSN is a highly ordered porous structure with no interconnection between individual porous channels. A high pore volume and large surface area of 1 cm^3^/g and 700 m^2^/g, respectively, avoids drug–drug interactions, and high drug adsorption facilitates fine control of drug loading and drug-release kinetics. The large pore volume facilitates cargo loading into micropore channels by enhancing drug–pore-wall intermolecular interactions. A tunable pore size between 50 to 300 nm favors optimal cellular uptake, enhanced circulation time, high drug loading, and accumulation in tumor tissue [79].

#### 3.4.2. Biocompatibility

The safety aspects are the major issue for nanoparticles with a high surface-to-volume ratio. The biocompatibility of pharmaceutical drug carriers is the prerequisite property to avoid the accumulation of carriers in the body. Although FDA has approved silica as GRAS (generally recognized as safe) for humans, assessment of the biocompatibility of MSNs is essential because the particle size, shape, dosage, and surface properties of MSNs are factors for its biodistribution and toxicity. The toxicity of silica nanoparticles is mostly associated with the cationic nature of the silanol group, which disturbs the membrane leading to cell lysis and subcellular component leakage [80]. Mesoporous silica showed very low hemolytic activity in vitro and non-lethal effects in mice during acute and sub-acute toxicity studies due to the low-density silanol group [81]. Repeated administration of hollow MSNs at a low dose causes no significant change in the hematological and biochemical parameters and nil morphological changes in the organ, suggesting MSNs are safe for oral and intravenous administration [82]. Surface functionalization improves the biocompatibility of MSNs for drug delivery.

#### 3.4.3. Biodistribution

Particle size, shape, and dimensions are important for nanoparticle distribution, circulation period, and clearance rate [72]. Short rod-shaped MSNs are concentrated in the liver, long rod-shaped in the spleen with a reduced elimination rate, and the rate of cellular entry by endocytosis is influenced by particle size [83].

#### 3.4.4. Biodegradability and Clearance

Generally, silica nanoparticles degrade into silicic acid (Si (OH)_4_) in a biological medium, which readily dissolves in water to form silicon species (<2 × 10^−3^ M) that are quickly excreted in the urine. The solubility of amorphous silica in an aqueous medium involves hydration in water to form a siloxane framework, which hydrolyses into silanols with subsequent formation of silicic acid, which leaks into blood/lymphatic circulation and clears in the urine. The degradation of MSNs is similar to that of silica nanoparticles [84]. Several studies evaluated the influence of size, shape, and morphology on the degradation and clearance of silica nanoparticles [85]. He et al. [86] analyzed MSNs of different diameters ranging from 80 to 360 nm, showing 45% excretion in the first 30 min with an enhanced clearance rate for larger particles. Nanoporous silica quantum dots of 3–6 nm were effectively excreted in two days; larger silica nanoparticles (20–25 nm) prefer hepatobiliary excretion within 15 days [87,88]. Cho et al. [89] reported nonporous silica nanoparticles dye-labeled with a size of 50 nm cleared faster than those of 100 and 200 nm diameter. Chen et al. observed the degradation of MSNs of different sizes in simulated body fluids (SBF) at 37 °C, which revealed that degradation is independent of size [90].

Around 45% degradation was observed in the first two days, with complete degradation within a week. Concerning the surface area, He et al. examined the degradation of MSNs of different surface areas in SBF sealed in polyethylene bottles at 37 °C subjected to mechanical shaking. The results showed fast degradation within the first 4 h with the highest silica hydrolytic degradation in increased surface area, and complete degradation was observed within 15 days [91]. The effect of morphology and aspect ratio on MSN degradation was assessed by incubating spherical and rod-shaped MSNs with different aspect ratios in simulated gastric fluid (SGF, pH 1.2), simulated intestinal fluid (SIF, pH 6.5), and SBF (pH 7) for 7 days. The results revealed no significant alteration in three MSNs; only 10% degradation was observed. However, in a complete DMEM medium, quick degradation of spherical-shaped MSN with aspect ratios of 2 and 4 was observed compared to nanorods. The surface modification also influences the clearance of MSNs [92]. Polyethylene glycol (PEF)-modified silica nanoparticles exhibited a higher circulation (t_1/2_—180 min) period when compared to unmodified (t_1/2_—80 min) and carboxylated silica nanoparticles (t_1/2_—30 min), as PEG provides stealthy behavior that prevents its uptake by reticuloendothelial cells and thereby increases its circulation time [76]. The elimination rate of MSNs increases with particle size affecting the degradation rate and biocompatibility [84]. 

## 4. MSNs: Theranostic Tool for Inflammatory Diseases

Generally, NPs interact with the body through various routes such as the skin, lungs, and gastrointestinal tract. Skin provides a larger surface area and easier accessibility for NPs [93]. A kind of non-specific interaction between the NPs and the plasma proteins produces a protein corona, which can partially compromise nanomedicine targeting [94] and affect the host immune activation. The unfolded protein corona activates reactive oxygen species (ROS) and proinflammatory cytokine production in murine and human macrophage cells. Certain in vivo studies have shown that the unfolded protein corona stimulates immune responses in the spleen, which is flooded with neutrophils, natural killer cells (NK cells), and CD8+ T cells. On the contrary, the normal protein corona with no structural changes does not create immune activation. The studies concluded that the highly unfolded protein corona can be involved in immune-enhancing therapies [95]. Inflammation naturally occurs as a protective response to external stimuli, infections, toxicity, injuries, or autoimmunity. Anomalous or prolonged inflammation can lead to various health implications such as diabetes mellitus, cardiovascular, neurodegenerative, atherosclerosis, pulmonary fibrosis, and many types of cancer [96,97,98,99,100].

### 4.1. Anti-Inflammatory Properties of MSNs

MSNs were used to limit inflammation in lipopolysaccharide (LPS)-induced rat macrophage cells. Most importantly, inflammation occurs through the arachidonic acid pathway with the help of the enzyme cyclooxygenase-I (COX-I) [101]. Caffeine loaded with MSNs (CMSN) and caffeine alone were studied using LPS-activated macrophage cells. When LPS-activated macrophages were exposed to both caffeine and CMSN, the CMSN significantly alleviated COX-2 and LOX-5 enzyme activity, reduced TNF-α expression, reduced the activities of the enzymes myeloperoxidase (MPO) and inducible nitric oxide synthase (iNOS), and ameliorated inflammatory signals compared to free caffeine. The study showed that MSNs could be a recommendable delivery system for components that bolster anti-inflammatory activities [102]. 

To evaluate inflammatory responses in myocardial infarction (MI) repair, Bao et al. developed engineered neutrophil apoptotic bodies (eNABs) to stimulate natural neutrophil apoptosis, regulate the inflammatory response, and enhance MI repair. eNABs and the neutrophil apoptotic body membrane (NABM) possibly decrease the pro-inflammatory cytokines TNF-α and IL-6 and stimulate TGF-β and IL-10 secretion. eNABs were prepared by fusing natural neutrophil apoptotic body membranes and MSNs preloaded with hexyl 5-aminolevulinate hydrochloride (MSN^HAL^). Thus, eNABs were highly suitable with inflammatory tropism and macrophage targeting ability, which carry the MSN^HAL^ nanovesicle into the infarcted heart. The study showed that MSN^HAL^ in the eNABs helps reprogram the macrophages, stimulating the anti-inflammatory effects and supporting cardiac function [103].

The anti-inflammatory responses of Fe/Ce-MSN and polyethylene glycosylated Fe/Ce-MS (Fe/Ce-MSN-PEG) nanoparticles were reported. Ferrite and ceria co-doped with MSN (Fe/Ce-MSN) and co-doped with PEG were prepared to study their ROS-scavenging and anti-inflammatory abilities in RAW 264.7 macrophage cells. Fe/Ce-MSN enhances the superoxide dismutase activities and exhibits anti-inflammatory activities. Fe/Ce-MSN-PEG significantly attenuated oxidative stress, inflammation induced by ROS, and macrophage apoptosis that occurs through high intracellular ROS and inhibited LPS-induced TNF-α and IL-1 β levels. Interestingly, Fe/Ce-MSN was more effective than Fe/Ce-MSN-PEG. The study proved that pH-responsive Fe/Ce-MSN and Fe/Ce-MSN-PEG enhance macrophage functions, protect the same from apoptosis, and facilitate anti-inflammatory nano therapies [104]. 

### 4.2. MSNs in Airway Inflammation

Severe lung inflammatory disease, such as acute lung injury (ALI), is characterised by increased lung permeability leading to alveolar edema [105], which activates the inflammatory response by releasing TNF-α, IL-6, and IL-1 β into the alveolar space of the affected lung [106]. Effective therapy for ALI depends on the specific delivery of drugs into the affected lungs. Recently, nanomaterials have been employed to deliver drugs to the affected lungs. MSNs were found to be suitable due to their excellent stability, high retention, and changeable pore sizes [107]. García-Fernandez studied the use of a nanodevice MSN loaded with rhodamine B (RhB) or dexamethasone (DEX) capped with a peptide that facilitates targeting of TNF receptor 1 (TNFR1), which is expressed on the surface of pro-inflammatory macrophages (TNFR-DEX-MSNs). The results showed that TNFR-DEX-MSNs bound with the pro-inflammatory M1 macrophages expressing TNFR and could internalize and reduce TNF-α, IL-1 β, and IL-6 levels in the lungs, with reduced side effects of DEX [108].

DEX is a well-known corticosteroid used to reduce inflammatory cells in the airways and enhance respiration [109]. The insoluble DEX can be combined with MSNs to increase its solubility. DEX loaded with MSPs showed high availability and ability to reach the lower part of the lungs. Melphalan (MEL)-induced airway inflammation can be treated with MSP-encapsulated DEX. In the LPS-induced inflammation model, the inflammatory markers such as myeloperoxidase (MPO), keratinocyte chemoattractant (KC), and matrix metalloproteinase-9 (MMP-9) were significantly reduced by empty MSNs; on the contrary, free DEX did not affect these inflammatory markers. The inhaled empty MSNs reduced inflammatory responses, irrespective of the presence of DEX, showing that the empty MSPs could also exert therapeutic functions. The mechanism still needs to be elucidated completely [110].

### 4.3. MSNs in Neuroinflammation

Neuroinflammation occurs due to any consequences or malfunctioning of the neurons. In neurodegenerative diseases such as AD, PD, and epilepsy, the BBB is disturbed, allowing inflammatory molecules through and causing neuronal inflammation and neural cell damage. Crossing the BBB and entering the neuronal environment is one of the major challenges in treating neuroinflammation [111].

The functionalization and shape of MSNs play a prominent role in crossing the BBB. Nonfunctionalized MSNs showed low permeability and cellular uptake, and PEG-PEI-functionalized MSNs showed improved cellular uptake without toxicity, indicating that MSNs are safe drug-delivery systems across the BBB [112]. The effect of MSNs and functionalized MSNs on cell viability, oxidative damage, and ROS formation were evaluated in rat pheochromocytoma PC 12 cells. Even prolonged exposure to MSNs at high concentrations did not affect the ROS formation and oxidative DNA damage; this shows that MSNs and functionalized MSNs are safe in PC 12 cells [113]. 

MSNs are used as a theranostic tool for intracerebral hemorrhage (ICH). ICH occurs due to a sudden rupture of the brain blood vessels. Inflammatory reactions at the perihematoma produce more damage to the brain resulting in increased ROS, oxidative stress, and neurodegeneration. Neuronal inflammation accompanies cellular components such as leukocytes, microglia, chemokines, cytokines, and ROS [114]. Lipid-coated MSNs are used as nanocarriers for ceria nanoparticles to scavenge ROS. Iron oxide NPs are used in MRI [115]. Lipid-coated MSNs possess anti-inflammatory and antioxidative effects. In rat ICH models, lipid-coated MSNs migrate to the perihematomal region and exert anti-inflammatory properties by destroying ROS and increasing the contrast of MRI. Lipid-coated MSNs reduce inflammatory macrophages and brain edema and support the sustainability of neuronal cells in ICH rats [111]. 

Spinal cord injury (SCI) is a neural inflammation condition characterized by an imbalanced inflammatory and oxidative condition of neurons, leading to delayed rebuilding or failure to rebuild the damaged neural networks. So far, hydrogen (H_2_) therapy can treat the spared neurons and allow the neuronal niche to develop new neuronal cells [116].

Hypoxia, hemorrhage, necrosis, neuronal cell death, inflammation, astrocyte activation, oxidative stress, and nerve conduction may occur during spinal cord transection [117]. Any secondary injury of SCI increases ROS levels and causes further degeneration of neurons and astrogliosis, resulting in a highly inflammatory environment [118]. Although H_2_ therapy is used to control oxidative stress and is found to be a promising way of alleviating inflammatory distress. Some difficulties, such as the optimal concentration and controlled release of H_2_ onto the injured area, are still challenging. To achieve effective delivery and release of H_2_, ammonia borane (AB) is used as a precursor for H_2_ production [119]. AB was assembled in MSNs (AB/mSiO_2_) and administered to SCI rats through intermittent intrathecal injection. After the release of H_2_ in the SCI region, its concentration and, its effects on motor functions, axonal regeneration, inflammatory modulation, and oxidative status were evaluated. The results showed that AB/mSiO_2_ enhanced motor function and neurofunctional recovery by facilitating neuronal protection and reduced SCI-induced scarring and astrogliosis. AB/mSiO_2_ administration significantly controlled the excess ROS and regulated acute inflammation. These results cumulatively show that AB/mSiO_2_ nanoparticles can be a potential therapeutic agent to treat neuronal inflammation after SCI [116]. 

Increased inflammatory responses caused by high levels of M1 macrophages are a characteristic feature of SCI. Interferon regulatory factor 5 (IRF-5), a protein involved in the macrophage phenotype change from M1 to M2, acts as a target in regulating the M1 to M2 phenotype programming. Polyethylenimine-conjugated, diselenide bridged MSNs are tailored with small interfering RNA (siRNA) to knock down IRF-5. MSNs act as an effective transfecting agent of SiRNA-IRF-5. MSNs release SiRNA-IRF-2, which regulates M1 to M2 macrophage conversion and effectively reduces excessive inflammation, promoting neuronal protection and motor function restoration [112]. ROS-responsive MSN-SiRNA inhibited the synthesis of TNF-α, IL-6, and iNOS, enhancing the anti-inflammatory cytokine (IL-10) in the SCI lesion. Thus, the ROS-responsive MSN improves the intracellular transfection rate, regulates the M1 and M2 macrophage transition, supports the anti-inflammatory responses, and promotes the neural protection and functional recovery of SCI mice [120]. 

### 4.4. MSNs in Inflammatory Bowel Disease

Inflammatory bowel disease (IBD) is an inflammatory condition of the gastrointestinal tract. Immune cells such as neutrophils, eosinophils, macrophages, and monocytes in the colon tissues are activated by injuries in IBD and initiate ROS, inflammatory factors, and proinflammatory cytokines. Targeting ROS and oxidative signaling remains the keyway to treat or prevent IBD [121]. The currently available treatments for IBD have disadvantages, including drug efficacy and adsorption in the small intestine. Different types of antioxidative nanoparticles have been developed to minimize or inhibit oxidative stress signaling and ROS, which support IBD treatment and prevention.

NPs are potent materials in targeted drug delivery to the intestine. Magnetic MSNs loaded with safranin O (S1) and hydrocortisone (S2), with azo derivatives containing urea moieties on the outer surface, were synthesized to release the entrapped drug into the colon region. The magnetic MSNs enhanced the retention time in the intestinal region. MSNs loaded with both S1 and S2 remained capped in the neutral pH. MSN-S1 delivery and binding were improved in rats wearing a magnetic belt. A Colitis rat model treated with MSN-S2 showed improved hydrocortisone retention. The study proved that MSNs could be used as a drug-delivery material into the intestine for treating IBD [122]. 

Similarly, MSN loaded with rhodamine B fluorophore (S1) and hydrocortisone (S2) capped with olsalazine derivative were studied in an ulcerative colitis rat model. The pharmacokinetic results revealed that S1 delivery is very specific to the colon. Treatment with S2 with a double-drug 5-ASA and hydrocortisone formulation in rats with chronic colon inflammation showed improved signs in pathology. This critically shows that MSNs could be used to deliver drugs to the colon region without being affected by absorption [123].

Materials such as damage-associated cell-free DNA (cfDNA) and ROS are crucial for mucosal damage and chronic inflammation in IBD cases [124]. Several internal and external stimuli can produce ROS, resulting in lipid peroxidation, apoptosis, and mucosal damage in the colonic region [125]. PEI coupled to antioxidative diselenide-bridged mesoporous organosilica nanoparticles (MON-PEI) exhibits cfDNA- and ROS-scavenging activity in mouse models of ulcerative colitis and Crohn’s disease. MON-PEI exhibits significant performance as a dual scavenger of cfDNA and ROS that aids in treating colitis. MON-PEI controls the colonial macrophage polarization by blocking cfDNA-induced TLR9-MyD88-NF-κB signaling and reducing the ROS-mediated pro-inflammatory response. The study results indicate that MON-PEI could attenuate peritoneal and colonic inflammation and prevent colon tissue damage [126]. 

### 4.5. MSNs in Arthritis Inflammation

Radial MSNs with protonated amines are the carrier for DEX to treat rheumatoid arthritis (RA)-associated inflammation. A rat model of RA treated with radial MSNs loaded with DEX showed significant anti-inflammatory effects and cartilage recovery. Radial MSNs provided highly efficient drug delivery and retained release of drugs in the affected knee region [127]. Osteoarthritis is a severe inflammatory condition characterized by lubrication failure and articular damage. MSNs have been developed to treat osteoarthritis by enhancing lubrication and local drug delivery. Biodegradable MSNs were synthesized using a biphasic oil-stratification method, later modified with poly (2-methacryloyloxyethyl phosphocholine) (PMPC) to produce lubricating drug-encapsulated nanoparticles (bMSNs-NH2@PMPC) for treating osteoarthritis. An in vitro degradation test showed that bMSNs-NH2@PMPC degraded within 7 days, and lubrication improved with a 50% reduction in the friction coefficient. bMSNs-NH2@PMPC showed sustained drug release without any toxic effects. These results substantiated that bMSNs-NH2@PMPC could be used to treat osteoarthritis [128].

Flurbiprofen (FLU; drug for arthritis) has a clinically very limited response due to its poor physicochemical and low soluble properties. A soluble and compatible drug carrier for FLU is necessary. Kondagogu gum (KG), a plant-derived rhamnogalacturonan from *Cochlospermum gossypium* [129], acts as an emulsifying gel and a good carrier for intragastric floating drug delivery [130]. FLU was loaded with KG emulgel reinforced with mesoporous calcium silicate to improve the gastroretentive ability and thermal stability. The drug-release ability of FLU-loaded matrices was studied. FLU entrapped with mesoporous silica composites showed an excellent drug release profile and in vitro gastro retention abilities. The mesoporous silicate-KG entrapped FLU emulgel system with enhanced biocompatibility is highly accessible and soluble and releases the drug in a controlled manner. The therapeutic functions of FLU were improved with minimal side effects and were effective in treating inflammation and arthritis [131]. 

### 4.6. MSNs as a Theranostic Tool for other Diseases

MSNs are widely used as carriers of drugs and delivery composites for different drugs for complex diseases related to anti-inflammatory effects, such as oxidative stress, microglial polarization regulation in the brain, wound healing as a tissue adhesive, atherosclerosis, corneal neovascularization, allergic inflammation, cancer, bone regeneration, lipid-lowering in obesity, ischemic stroke, etc. The functional attributes and findings of MSNs as nanocomposites in various diseases are detailed in Table 2.

## 5. Limitations

The intracellular delivery of MSNs to specific cells to control cell signaling is challenging. The biological membrane acts as a barrier to intracellular delivery, so the MSN drug carrier must be designed to cross the biological membranes to attain the specific drug delivery. Several factors, including size and morphology, surface functionalization [145], and electrostatic interaction between the cell membrane and MSNs affect the internalization of MSNs [80]. The particle size influences the internalization process. If the size of MSNs is <200–300 nm, they can be taken up by the cells via the endocytosis pathway [80,146]. Even though studies claim that MSNs can easily internalize into the target cells [147,148], in some cases, for example, MSNs have not induced sufficient osteogenic signals in human mesenchymal stem cells (hMSCs) [149].

The high reactive porosity in the MSNs might induce nanotoxicity in addition to their biological efficiencies in cellular uptake and immune activation. As far as the advantages are concerned, MSNs were detrimental to skin types weakened by AD conditions. MSNs can penetrate deep into the skin of AD patients and form a protein corona in the skin and body fluids, which eventually causes trouble to immune homeostasis resulting in severe and more aggravated AD and inflammation [150]. Encapsulating drugs with MSNs to treat lung injury possesses certain limitations, such as designing silica NPs embedded with drugs of the proper dosage and delivery to targeted inflamed lungs to minimize the DEX-derived side effects [108].

Asthma is one of the chronic allergy diseases of the airway induced by certain environmental pollutants and nanoparticles [151]. The effects of three kinds of SNPs, the spherical SNPs (S-SNPs), MSPs, and PEG-SNPs, were evaluated in the ovalbumin-induced allergic airway inflammation in 6-week-old BALAb/c mice [152]. Han et al. showed that, even though the SNPs are safe and have been used in various applications such as cosmetics and drug-delivery systems, exposure to SNPs with or without certain pollutants and allergens might induce the allergic mechanism in the respiratory system. The inhalation of SNPs induces toxic effects and produces airway hyperresponsiveness (AHR), increasing the inflammatory cells in bronchoalveolar lavage fluid (BALF) and cytokine levels in the lungs. Compared to the spherical SNPS, MSPs, and PEG-MSNs, the MSNs significantly induce airway inflammation through increased AHR and cytokines (IL-5, IL-13, IL-1β, and IFN-γ), in addition to the induced AHR and increased interleukin levels [152]. Likewise, the PEG-SNPs also induce lesser inflammation compared to MSN and S-SNPs. The study indicates that the larger surface area of MSNs could be a limitation of these particles in inducing severe inflammation compared to other SNPs. Thus, exposure to MNPs can also induce toxicity and airway inflammation diseases and procuring more safety measurements while using MSNs is required [152].

In vivo aggregation of MSNs in vital human organs such as the liver, spleen, and bladder produces serious illness such as inflammatory reactions, oxidative damage, and organ fibrosis [153]. In osteoarthritis, MSNs are difficult to degrade in a short duration in vivo. On this occasion, MSN bioaccumulation might induce toxicity in the localized tissue and ultimately enhance inflammation. For such reasons, it was considered to develop biodegradable MSNs with the help of the oil–water biphase method [128]. Designing biocompatible and multifunctional MSNs and avoiding MSN bioaccumulation in target sites have been the strongest research areas in nanomedicine. Although SNPs are considered safe and less toxic, there are serious questions regarding their safety and biocompatibility in the brain when used in neuronal inflammatory diseases. The biocompatibility of MSNs can be affected by their physical properties, such as particle size, surface properties, and shape. The toxicity of any functionalized nanoparticle also depends on the dosage. Nanoparticles with several silanol groups induced high ROS and became toxic, but MSNs showed lower cytotoxicity and haemolytic activity due to their fewer cell contact regions [111].

## 6. Conclusions

Employing nanoscale materials as biological tools in diagnostic and treatment platforms keeps developing. Despite the growing need for eminent diagnosis and therapy, single or combined therapeutic and diagnostic methods have become a prerequisite for disease management. Emerging theranostic tools constructed with different biocompatible nanoparticles are of great scientific interest. They are tested against inflammatory diseases, wound healing, cancer, cardiovascular diseases, pulmonary fibrosis, and neuroinflammatory diseases. The discovery of MSNs with superior physicochemical properties has become a promising tool in biomedical applications as nano drug carriers and delivery systems. Recently several kinds of nanoparticles have been used in drug delivery of neuroactive molecules to the brain by crossing the BBB due to the clinical advantages of nanoparticles in minimal drug dose, with reduced or no side effects, longer shelf life, and increased success rate in entering the BBB. However, the nanotechnology-based approaches in CNS applications remain to be answered and require more preclinical studies for treating neuroinflammatory diseases. Despite the safety of MSNs, there are still numerous questions related to dosage, bioaccumulation, and removal of MSNs from the body. Thus, further detailed studies are needed on the optimization and safety of MSN-based drug-delivery systems to treat diseases and disorders effectively.

## Figures and Tables

**Table 1:** Types of MSN used for drug delivery applications.

S. No.	MSN Family	Type	Pore Symmetry	Pore Size (nm)	Pore Volume (cm^3^/g)	Application	Ref.
1	M41S	MCM-41	2D hexagonal *P6mm* unidirectional	1.5 to 8	>10	Drug delivery, adsorbent, catalysis, biosensor	[38]
		MCM-48	3-D cubic *Ia3d*	2 to 5	>10		[39]
		MCM-50	3D lamellar *P2*	2 to 5	>10	Excellent adsorbent and catalysis	[40]
2	SBA	SBA-11	3-D cubic *Pm3m*	2.1 to 3.6	0.68		[41]
		SBA-12	3D hexagonal *P6_3_/mmc*	3.1	0.83		[42]
		SBA-15	2D hexagonal *p6mm*	6	1.17	Drug delivery, adsorbent, catalysis, biosensor	[42]
		SBA-16	Cubic *Im3m*	5 to 15	0.91		[43]
3	KIT	KIT-5	Cubic *Im3m*	9.3	0.45	-	[42]
4	COK	COK-12	Hexagonal *P6m*	5.8	0.45	-	[44]
5	Modern MSN	Hollow MSN	-	-	-	-	[45]

MCM: Mobile crystalline materials; SBA: Santa Barbara amorphous; KIT: Korean Advanced Institute of Science and Technology; COK: Centre for Research Chemistry and Catalysis.

**Table 2:** Theranostic properties of MSNs in various diseases. All the studies are in the preclinical stage. The information on regulatory approval and clinical trials needs to be available.

Nanocomposite and Drug	Model	Results Obtained	Ref.
Anti-oxidant property			
Anionic mesoporous silica-6 (AMS-6) with Probucol (PB)	Zebrafish and human brain microvascular endothelial cells	Enhanced solubility and release of PB from AMS-6. Mitigated the effects of oxidative stress by inhibiting mitochondrial hydroxyl radical generation and COX-2-mediated PGE_2_. Reduced ROS produced by LPS.	[132]
Mesoporous silica-coated cerium oxide (CeO_2_) nano enzyme	Obese Zucker rats and human hepatic cell line (HepG2 cells).	Maximized ROS-scavenging activity and improved anti-inflammatory effects. Improved metabolic profile of obese rats. Ameliorated hyperlipidemia and hepatic and metabolic dysregulation. Reduced M1 macrophages and TNF-α.	[133]
Corneal neovascularization			
Mesoporous silica nanoparticles loaded with anti-VEGF bevacizumab (BEV) incorporated into cyclosporin A (CSA) thermo gel. (BEV@MSN-CsA@Thermogel)	Male New Zealand white rabbits, primary human tenon’s fibroblasts, human corneal epithelial cells, human corneal endothelial cells, and human umbilical vein endothelial cells.	BEV@MSN-CsA@Thermogel regulated the release of BEV and CSA in a suitable ratio sustained for up to 4 weeks. Minimal toxicity and high biosafety in vivo. Showed the highest inhibitive effect on VEGF-induced HUVECs in terms of their proliferation, migration, and tube formation. Inhibited corneal neovascularization in terms of the CNV area, new vessel length, corneal opaque, corneal inflammation, and abnormal fibrosis in a rabbit model. Enhanced CNV inhibition through synergistic anti-VEGF and anti-inflammatory effects.	[134]
Wound healing			
Three-dimensional (3D) dendritic mesoporous silica nanoparticles	Sprague Dawley Rats	MSN dispersion and degradation allow wound tissue to adhere fast. MSNs blend with wound exudates in the injury and form interconnected organic-inorganic nanocomposites. MSNs generated a fibrin matrix for innate wound closure and recruited inflammation as the bioactive scaffold. Elimination of MSN, reconstruction and inflammatory response resolved, skin integrity restored, new epithelium generated.	[135]
Poly (lactic-co-glycolic acid) nanofibrous wound dressing incorporated with andrographolide-loaded mesoporous silica nanoparticles (PLGA/Andro-MSNs).	*Staphylococcus aureus*-infected wound mice and human keratinocyte cell line (HaCat)	MSNs and Andro nanoparticles might promote cell adhesion, migration, and proliferation. MSNs promote epidermal cell adhesion and prolong the Andro effects. MSNs slow drug release and convert crystalline Andro into a noncrystalline state. Andro weakened the inflammatory activities at the wound site. PLGA/Andro-MSNs enhanced epidermal cell adhesion, reduced inflammation, and exhibited efficient wound-healing activity.	[136]
A core-shell structured silver core embedded with mesoporous silica-based nanoplatform co-load with ciprofloxacin and TNF-α and Si TNF-α (AMPC@siTNF-α)	BALB/c mice and RAW 264.7 macrophage cells from immortalized mouse myoblast cell line	AMPC@siTNF-α-treated group showed no inflammation or scabs after 8 d of treatment, and the wound completely closed at 12 d in vivo. AMPC@siTNF-α showed controlled release of Ag+, antibiotics, and SiRNA and exhibited antibacterial activity in vitro. AMPC@siTNF-α internalizes macrophages and reduces expression of inflammatory factors TNF-α in vitro.	[137]
MSN decorated with Ceria (MSN-Ceria)	Male Sprague Dawley rats and human skin keratinocytes (HaCaT cells)	Possess strong tissue adhesion strength. Significantly reduced ROS-mediated effects. Accelerated wound healing process and regenerative capacity. Highly developed skin appendage morphogenesis and limited scar formation.	[138]
Photocurable methacryloxylated silk fibroin hydrogel (Sil-MA) system, co-encapsulated with metformin-loaded mesoporous silica microspheres (MET@ MSNs) and silver nanoparticles (Ag NPs).	Streptozotocin (STZ)-induced diabetic C57BL/6 mice, EA. vy926, L929, RAW264.7 cells	Enhanced wound-healing ability through spatiotemporal immunomodulation. Controlled and sustained release of Ag NPs help inhibit bacterial aggregation and maintain a sterile microenvironment. The M1 phenotype of macrophages in regions of diabetic trauma shifted to an anti-inflammatory M2 phenotype. Inhibited the formation of neutrophil extracellular traps (NETs) and decreased the release of neutrophil elastase, myeloperoxidase, and NETs-induced pro-inflammatory factors. Improved fibroblast migration and endothelial cell angiogenesis in vivo, with enhanced diabetic-wound healing.	[139]
Ischemic stroke			
Lactoferrin functionalized hollow mesoporous manganese doped silica nanoparticles (LHMMSN) loaded with resveratrol (RES)	Sprague Dawley rats and rat nerve cells (PC12), mouse brain microvascular endothelial cells (bEnd.3 cells) and mouse microglial cells (BV2)	In vivo imaging showed that the fluorescence intensity of brain hemispheres in MCAO rats suggested that LHMMSN could cross the BBB. Improved the water solubility and dissolution of RES. Bioavailability and efficacy of RES improved. LHMMSN-RES effectively inhibit oxidative stress (ROS, MDA) and increase the content of SOD and GSH to protect neurons. Increased expression of anti-inflammatory factors and anti-apoptotic factors in brain tissue. Reduced brain tissue pro-inflammatory factor (TNF-α, IL-1b, IL-6) and pro-apoptotic factor (BAX, Cleaved caspase-3) expression, reduced inflammation, and protect nerve cells by inhibiting oxidative stress. Promoted the recovery of motor function in MCAO model rats.	[140]
Atherosclerosis			
MSNs were loaded with the lipid-lowering drug simvastatin (SIM) and gated with hyaluronic acid (HA) coating (SIM@HA-MSN).	Male C57BL/6 mice, mouse macrophages (Raw264.7 cells), and human umbilical vein endothelial cells (HUVECs)	SIM@HA-MSN achieved satisfactory functionalization with HA provided with high loading efficiency, lipid-lowering effect for treating atherosclerosis HA conjugation reduced the premature release of drugs and support. SIM@HA-MSN attenuated the production of TNF-α and IL-6 and prevented the foaming of macrophages. Enhanced cyto/hemo compatibility and prolonged plasma retention time of MSNs, help treat vascular disorders, including atherosclerotic plaques. SIM@HA-MSN can be potentially safe and effective in combating atherosclerosis with its controlled drug delivery, anti-inflammation and anti-foaming efficacy, potent targeting ability, and good biocompatibility.	[141]
IL-1 receptor antagonist (IL-1Ra) loaded copper doped MSNs (IL-1Ra@Cu-MSNs)	Healthy C57BL/6 male mice, Apolipoprotein E knockout (ApoE−/−) mice and Raw264.7 macrophage cells	In the co-delivery nanoplatform, IL-1Ra was used as an IL-1 receptor-targeting and anti-inflammatory agent, and copper ions were used to trigger intracellular ROS production. Cu-MSN showed high drug loading efficiency, sustained release, and biodegradable ability. Released copper ions specifically induced macrophage apoptosis by triggering ROS production. Reduced lipid deposition and macrophage infiltration in a murine model of atherosclerosis. IL-1Ra@Cu-MSNs significantly reduced arterial stenosis, plaque burden and macrophage infiltration due to the combined action of copper ions and IL-1Ra.	[142]
Induced inflammation			
House dust mite (HDM) allergen *Dermatophagoides farina* and *Dermatophagoides* (Der f2) loaded with hollow MSN (HMSN)	Pathogen-free female BALB/c mice	Slow in vitro release of Der f2 from HMSNs help as an adjuvant and a delivery vehicle with bound protein remaining stable and biologically functional. Reduced Levels of Der f2 sIgE, a pathogenic antibody isotype for allergy in mice treated with both Der f2-HMSNs and Der f2/Al (OH)_3_. Der f2-HMSNs could decrease IL-4 levels and increase IFN-γ levels in the BALF of allergic mice. Der f2-HMSNs prevent the pathological Th2-response to Der f2, which indicates that HMSNs may be potential adjuvants for Der f2 protein in specific immunotherapy.	[143]
Dendritic MSNs with sustained H_2_S delivery system consisted of S-propargyl-cysteine (SPRC@DMSN)	Adjuvant-induced arthritis (AIA) rat model and bone-marrow-derived monocytes isolated from healthy mice	Significantly alleviated the symptoms and prevented bone damage by elevation of endogenous H_2_S. DMSN could be a novel drug carrier with low toxicity and high biocompatibility for treating arthritis.	[144]
Cancer			
Hollow mesoporous silica nanoparticles (HMSNs) with transferrin (Tf) targeting moieties	MDA-MB-231 cell lines, RAW264.7 macrophage cells	HMSN with disulphide linkage (HMSN-S-S-Tf) carriers were selectively enriched at the target tumor site and efficiently internalized by the cancer cells. Sustained and effective tumor suppression. Tf corona moiety enhanced the nanocomposite circulation in the blood and alleviated inflammatory responses.	[79]
Mesoporous silica nanoparticles loaded with anti-tumor component harmine (HM@MSN)	Lymphoma model of Balb/c mice, human lymphoma cell line Daudi, Raw264.7 cells	HM@MSN remained nontoxic with effective anti-lymphoma drug delivery ability and inhibited the formation of lymphoma clones. Downregulated the expression of NF-κB p65, TNF-α, and IL-6 genes, thereby suppressing tumor cell survival. Upregulated lymphoma cell apoptosis.	[55]

AMS-6: Anionic mesoporous silica particles; PB: Probucol; COX-2: Cyclooxygenase 2; PGE_2_: Prostaglandin E2; ROS: Reactive oxygen species; LPS: Lipopolysaccharide; TNF-α: Tumour necrosis factor-α; CNV: Corneal neovascularization; VEGF: Vascular endothelial growth factor; HUVECs: Human umbilical vein endothelial cells; EA.vy926: Hypomorphic human endothelial cell; L929: Mouse fibroblast cell line; M1, M2: Phenotypes of macrophages; NETs: Neutrophil extracellular traps; MCAO: Middle cerebral artery occlusion; sIgE: Serum IgE; Th2: T helper 2 cells; H_2_S: hydrogen sulfide.

## Data Availability

Not applicable.
